# IL-5 and IL-6 are increased in the frontal recess of eosinophilic chronic rhinosinusitis patients

**DOI:** 10.1186/s40463-017-0214-2

**Published:** 2017-05-02

**Authors:** Kazunori Kubota, Sachio Takeno, Takayuki Taruya, Atsushi Sasaki, Takashi Ishino, Katsuhiro Hirakawa

**Affiliations:** Department of Otolaryngology, Head and Neck Surgery, Division of Clinical Medical Science, Programs for Applied Biomedicine, Graduate School of Biomedical Sciences, Hiroshima University, 1-2-3 Kasumi, Minami-ku, Hiroshima, 734-8551 Japan

**Keywords:** Frontal recess, Eosinophilic chronic rhinosinusitis, IL-5, IL-6

## Abstract

**Background:**

Eosinophilic chronic frontal sinusitis is difficult to treat compared with non-eosinophilic sinusitis because of recurring inflammation and polyp formation in the frontal recess after the post-operative follow-up period. Studying inflammatory mediators in the frontal recess of eosinophilic chronic rhinosinusitis (ECRS) patients and non-eosinophilic chronic rhinosinusitis (non-ECRS) patients may lead to a better understanding of the pathogenesis of chronic frontal sinusitis.

**Methods:**

Homogenates of sinonasal mucosa from 20 non-ECRS patients and 36 ECRS patients were measured for levels of transforming growth factor (TGF)-β, interleukin (IL)-5, IL-6, and inducible nitric oxide synthase (iNOS) using real-time RT-PCR and TaqMan gene expression assays. Sinonasal mucosal specimens were obtained from the frontal recess, ethmoid sinus, and nasal polyp separately.

**Results:**

The expression of IL-5 was significantly elevated in all sinonasal regions tested in the ECRS group, but absent in non-ECRS patients. Furthermore, the ECRS patients showed significantly increased levels of IL-5 in the frontal recess mucosa compared with ethmoid sinus mucosa. IL-6 was also significantly increased in the frontal recess mucosa compared with ethmoid sinus mucosa and nasal polyps in these patients. There were no significant differences in the levels of TGF-β or iNOS between the ECRS and non-ECRS groups in any sinonasal region tested.

**Conclusions:**

This study is the first to characterize the cytokine milieu in the frontal recess of ECRS patients. We should keep these cytokine profiles in mind when we treat ECRS patients with frontal sinusitis.

## Background

Chronic frontal sinusitis is a complex disease for rhinologists to treat because of the difficulty in complete dissection of frontal recess cells and recurring inflammation. Even if the surgeon could remove all frontal recess cells that compromise the ventilation pathway, damage to the frontal sinus in the operation, such as bone exposure caused by mucosal damage, and a narrow ventilation pathway can lead to the restenosis of the frontal recess. Thus, frontal sinusitis recurs during the post-operative follow-up period [1]. The Modified Lothrop procedure (MLP) or Draf type 3 frontal drillout is often chosen to gain a wider ventilation pathway to the frontal sinus if the patient is considered at high risk of recurrence [2].

Surgical intervention is therefore essential for the treatment of chronic frontal sinusitis, however, there is a growing evidence base that emphasizes the importance of also understanding the pathophysiology of chronic rhinosinusitis [3].

Eosinophilic chronic rhinosinusitis (ECRS) is a subtype of recalcitrant chronic rhinosinusitis (CRS). Diagnostic criteria were established in 2015 by the Japanese Epidemiological Survey of Refractory Eosinophilic Chronic Rhinosinusitis study (JESREC) in Japan [4]. ECRS is characterized by marked infiltration of eosinophils in the paranasal sinus mucosa. Ostiomeatal complex occlusion may not be a predisposing factor for the development of ECRS, but the degree of mucosal eosinophilic infiltration and subsequent inflammation is important. Therefore, for the treatment of frontal sinusitis in ECRS patients, it is crucial to know the local cytokine profiles surrounding the frontal recess. The cytokine profile of the ethmoid sinus mucosa and nasal polyps in ECRS or chronic rhinosinusitis with nasal polyp (CRSwNP) patients has been reported previously. Ethmoid sinus mucosa of CRSwNP patients showed significantly increased expression of interleukin (IL)-5, eosinophil-cationic protein, immunoglobulin E (IgE), and *Staphylococcus* enterotoxin (SAE)-IgE compared with chronic rhinosinusitis without nasal polyp (CRSsNP) patients [5, 6]. Ethmoid sinus mucosa of ECRS patients also showed significantly elevated expression of inducible nitric oxide synthase (iNOS) and IL-5 [7, 8]. However, there is no report that investigates the cytokine profile in the frontal sinus mucosa of ECRS patients. In this study, we examined the cytokine profile in the frontal recess of ECRS patients to further elucidate the pathogenesis of the disease. These findings may lead to the prevention of recurrence of inflammation or nasal polyp.

## Methods

### Patients

Sinonasal mucosa from Japanese patients with ECRS (*n* = 36) and non-ECRS (*n* = 20) was obtained during endoscopic sinus surgery (ESS) at Hiroshima University Hospital from July 2010 to November 2016. Nasal polyps and sinus mucosa from the frontal recess and the ethmoid sinus were harvested separately. Nasal polyps were obtained from the middle meatus, and ethmoid mucosa was obtained mainly from the anterior ethmoid sinuses. Frontal recess mucosa was obtained from as near as possible to the frontal sinus, and included frontal cells and frontal bullar cells. The patients were routinely questioned regarding rhinosinusitis symptoms and received ear, nose and throat examination by flexible endoscopy. Criteria for ECRS from the JESREC study were adopted to distinguish ECRS patients from non-ECRS patients [4]. Computed tomography (CT) scan images of paranasal sinuses were evaluated by the Lund and Mackay scoring system [9]. Peripheral blood samples were obtained from all patients to measure blood eosinophil count and serum IgE levels.. Atopic status was considered positive if the patient had radioallergosorbent test (RAST) scores greater than 2 for any inhaled allergen. These allergens included Japanese cedar, Japanese cypress, house dust mite, common ragweed, orchard grass, and *Aspergillus*. Diagnosis of bronchial asthma was made by a pneumologist based on lung function and challenge tests, where applicable. Patients who received oral or intranasal topical steroids within 4 weeks of the surgery were excluded from the study.

The study protocol was approved by the Institutional Review Board at the Hiroshima University School of Medicine (approval number Hi-136) and written informed consent was obtained from all patients prior to inclusion in the study.

### Real-time RT-PCR analysis

Obtained tissue specimens were either minced with scissors immediately after surgery and immersed in RNAlater™ solution (Ambion, Austin, TX, USA) for real time RT-PCR analysis or fixed in 4% paraformaldehyde for immunohistochemistry. We eliminated frontal recess mucosa and nasal polyp specimens which were thought to be too small or damaged by during surgery. In total, we processed 21 frontal recess mucosa, 36 ethmoid mucosa, and 30 nasal polyp samples from 36 ECRS patients and 10 frontal recess mucosa, 20 ethmoid mucosa, and 9 nasal polyp samples from 20 non-ECRS patients.

Cellular RNA was isolated using RNeasy mini kits (Qiagen, Valencia, CA, USA). Total RNA was then reverse-transcribed to cDNA using a High Capacity RNA-to-cDNA kit (Applied Biosystems, Foster City, CA, USA) per the manufacturer’s instructions. Gene expression was measured on an ABI Prism 7300 system (Applied Biosystems) using TaqMan Gene Expression Assays. PCR primers specific for TGF-β (Hs99999918_m1), IL-5 (Hs00174200_m1), IL-6 (Hs00985639_m1), iNOS (Hs01075529_m1) and GAPDH (Hs99999905_m1) were used. GAPDH was used as a reference gene. PCR assays were run in triplicate for each sample. Amplifications of the PCR products were quantified by the number of cycles and results were analyzed using the comparative cycle threshold (Ct) method (2^−ΔΔ^Ct). The Ct values for target genes were normalized to the value of GAPDH by calculating the change in Ct (^Δ^Ct). Ct values of 34 or higher were considered as the lowest limit of detection. The quantities of target gene expression are presented relative to the expression of the reference gene (ratio: target gene/GAPDH expression).

### Immunohistochemistry

Immunostaining was carried out on 5-μm-thick cryostat sections of mucosal specimens. The anti-human IL-5 rabbit polyclonal antibody was from Acris (Rockville, MD, USA) and IL-6 rabbit polyclonal antibody was from GeneTex (Irvine, CA, USA). For antigen retrieval, sections were immersed in Histo VT One (Nacalai Tesque, Kyoto, Japan) at 70 °C for 40 min. The sections were then incubated overnight at 4 °C in the presence of the primary antibodies. Color development was performed using the streptavidin-biotin amplification technique (ChemMate En Vision kit, Dako, Glostrup, Denmark). Control specimens developed without the primary antibody were used to verify the absence of nonspecific binding. Consecutive sections were stained with hematoxylin-eosin (HE) to view the mucosal pathology and assess the degree of eosinophil infiltration.

### Statistical analysis

Statistical analyses were performed using Excel Statistics 2010 (Shakai Jouhou Corp., Tokyo, Japan). Baseline characteristics were compared with the Mann-Whitney *U* test between the non-ECRS and ECRS patients. Data are expressed as the mean and range. For the RT-PCR analysis, the Mann-Whitney *U* test was used to compare the groups. A value of *p* < 0.05 was considered statistically significant for all measurements.

## Results

### Comparison of clinical characteristics of ECRS and non-ECRS patients

The clinical characteristics of patients in this study are shown in Table 1. The average age of the ECRS patients was significantly younger than the non-ECRS patients. The atopic status and serum IgE levels did not show a significant difference between the non-ECRS and ECRS groups, whereas the peripheral blood eosinophil count was significantly higher in the ECRS group as expected. Sinonasal findings including bilateral lesion, nasal polyps, and predominant opacification of the ethmoid sinus were significantly worse in the ECRS patients. CT scores of the ethmoid and frontal sinuses were significantly higher in the ECRS patients. A significantly higher frequency of comorbid asthma was reported in ECRS patients.

**Table 1:** Baseline characteristics of the study population

	non-ECRS	ECRS	*P* value
Number of patients	20	36	-
Age(years old; range)	64.7 (39–76)	56.1 (23–74)	0.009
Female/male	5/15	11/25	N.S.
Atopic status(over class 2)	9/20	20/36	N.S.
Serum IgE(IU/ml)	424.5 (13–3767)	441.4 (8.6–3700)	N.S.
Peripheral blood eosinophil count(%)	3.0 (0.6–8.5)	8.5 (1.8–21.7)	<0.001
Bilateral lesion	10/20	35/36	<0.001
Nasal polyps	11/20	34/36	<0.001
Predoninant opacification of the ethmoid sinus	1/20	27/36	<0.001
Mean ethmoid score	1.23 (0–4)	3.02 (1–4)	<0.001
Mean frontal score	1.20 (0–3)	2.28 (0–4)	0.012
Comorbidity of Asthma	1/20	13/36	0.010
Comorbidity of otitis media	0/20	5/36	N.S.

Data are shown as the mean with ranges in parentheses. The level of significance was obtained by the Mann-Whitney *U* test

### IL-5 and IL-6 expression are increased in the frontal recess mucosa of ECRS patients

**Fig. 1:**
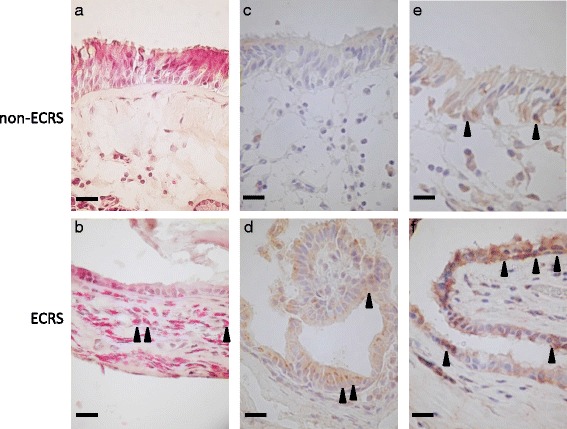
Histopathological features and cytokine expression in the frontal recess of ECRS and non-ECRS patients. Histopathological findings of frontal recess mucosa from non-ECRS patients (**a**, **c** and **e**) and ECRS patients (**b**, **d** and **f**). Hematoxylin-eosin staining (**a** and **b**), and immunohistochemistry (**c** and **d** show IL-5 positive cells, E and F show IL-6 positive cells) of patient samples. *Arrow heads* point to positive cells. Scale bars: 20 μm. Magnification: ×400

Immunohistological staining showing the distribution of IL-5 and IL-6 positive cells in the frontal recess mucosa, with corresponding H&E staining is shown in Fig. 1. The ECRS patients demonstrated greater accumulation of eosinophils in the submucosal layer as compared with the non-ECRS patients (Fig. 1a, b). The frontal recess mucosa of the ECRS patients also tended to show stronger staining for IL-5 (Fig. 1c, d) and IL-6 (Fig. 1e, d) in the epithelial cell cytoplasm and submucosal gland cell cytoplasm as compared with the non-ECRS patients.

**Fig. 2:**
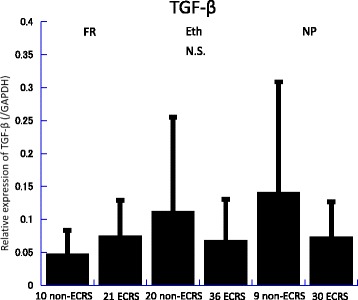
Measurement of TGF-β in 20 non-eosinophilic chronic rhinosinusitis (non-ECRS) patients and 36 eosinophilic chronic rhinosinusitis (ECRS) patients by RT-PCR. Data are presented as the mean values and error bars indicate standard deviation. FR = frontal recess; Eth = ethmoid sinus; NP = nasal polyp; N.S. = not significant

**Fig. 3:**
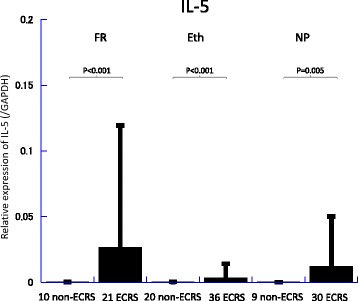
Measurement of IL-5 in 20 non-eosinophilic chronic rhinosinusitis (non-ECRS) patients and 36 eosinophilic chronic rhinosinusitis (ECRS) patients by RT-PCR. IL-5 is significantly increased in the frontal recess, ethmoid sinus and nasal polyp of patients with ECRS compared with non-ECRS. Data are presented as the mean values and error bars indicate standard deviation. FR = frontal recess; Eth = ethmoid sinus; NP = nasal polyp

**Fig. 4:**
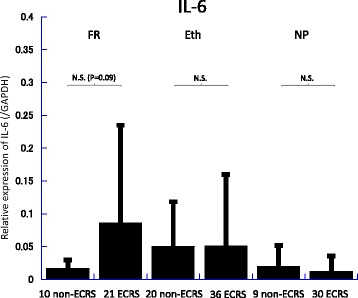
Measurement of IL-6 in 20 non-eosinophilic chronic rhinosinusitis (non-ECRS) patients and 36 eosinophilic chronic rhinosinusitis (ECRS) patients by RT-PCR. Data are presented as the mean values and error bars indicate standard deviation. FR = frontal recess; Eth = ethmoid sinus; NP = nasal polyp; N.S. = not significant

**Fig. 5:**
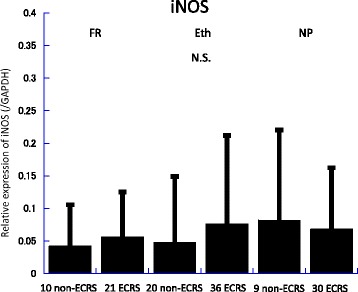
Measurement of iNOS in 20 non-eosinophilic chronic rhinosinusitis (non-ECRS) patients and 36 eosinophilic chronic rhinosinusitis (ECRS) patients by RT-PCR. Data are presented as the mean values and error bars indicate standard deviation. FR = frontal recess; Eth = ethmoid sinus; NP = nasal polyp; N.S. = not significant

**Fig. 6:**
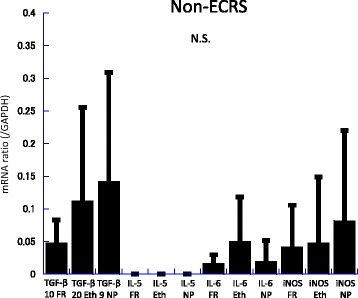
Measurement of TGF-β, IL-5, IL-6, and iNOS in 20 non-eosinophilic chronic rhinosinusitis (non-ECRS) patients by RT-PCR. In non-ECRS patients, there are no significant difference of any cytokine expression among frontal recess mucosa, ethmoid sinus mucosa and nasal polyp. Data are presented as the mean values and error bars indicate standard deviation. FR = frontal recess; Eth = ethmoid sinus; NP = nasal polyp; N.S. = not significant

**Fig. 7:**
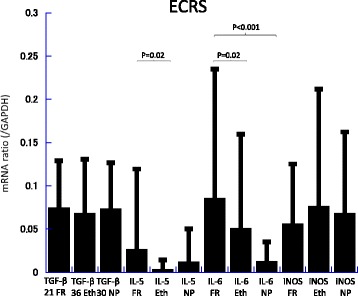
Measurement of TGF-β, IL-5, IL-6, and iNOS in 36 eosinophilic chronic rhinosinusitis (ECRS) patients by RT-PCR. In ECRS patients, frontal recess mucosa showed significantly higher expression of IL-5 and IL-6 compared with ethmoid sinus mucosa. Data are presented as the mean values and error bars indicate standard deviation. FR = frontal recess; Eth = ethmoid sinus; NP = nasal polyp

Messenger RNA (mRNA) levels of TGF-β, IL-5, IL-6, and iNOS in the frontal recess, ethmoid mucosa and nasal polyps were assessed by quantitative RT-PCR analysis (Figs. 2, 3, 4, 5, 6 and 7). There was no significant difference in mRNA levels of TGF-β (Fig. 2), IL-6 (Fig. 4) and iNOS (Fig. 5) between the two groups in any sinonasal region; frontal recess mucosa, ethmoid sinus mucosa, or nasal polyps. However, ECRS patients showed significant upregulation of IL-5 mRNA compared with non-ECRS patients in all sinonasal regions, with the increase being more prominent in the frontal recess and nasal polyps (Fig. 3). Moreover, in ECRS patients, frontal recess mucosa showed significantly higher levels of IL-5 mRNA compared with ethmoid sinus mucosa. Additionally, frontal recess mucosa showed significantly higher levels of IL-6 compared with ethmoid sinus mucosa and nasal polyp (Fig. 7). In non-ECRS patients, no such specific profile of cytokine expression was observed among sinonasal regions (Fig. 6).

## Discussion

There is growing evidence that not all CRS is a simple disease treated by medication and ESS [3]. Notably, the number of CRS patients who have recurrence of nasal polyposis after the initial ESS and need revision surgery is increasing [2]. Based on this background, a scoring systems for refractory chronic rhinosinusitis, also called ECRS in Japan, was established in 2015. This system enabled us to easily classify CRS patients into two groups and to compare various clinical parameters between them [4].

ECRS is characterized by high eosinophil counts, significant inflammation in the ethmoid sinus, and inflammatory lesions that also includes the frontal recess. Findings such as higher eosinophil counts and predominant ethmoid inflammatory disease are not surprising in ECRS patients as they are part of the diagnostic criteria [4]. MLP or Draf type 3 frontal drillout have become popular in treating recalcitrant frontal sinusitis. However, post-operative frontal ostium restenosis associated with accumulation of eosinophilic mucin is sometimes inevitable in ECRS patients [10].

We previously investigated the relationship between the prevalence of frontal sinusitis and the radiological features of frontal recess cells [11]. In the present study, we have extended this to examine the cytokine profile of frontal recess mucosa that underlies the prolonged inflammatory processes. For this purpose, we selected three representative cytokines (TGF-β, IL-5, IL-6) and one enzyme (iNOS) that may discriminate between the pathophysiology of ECRS and non-ECRS.

It is widely accepted that the cytokine expression in the paranasal mucosa of CRSwNP patients differs from that of CRSsNP patients [6, 12, 13]. In fact, nasal polyp tissue, when compared with adjacent inflammatory nasal mucosa, was shown to express higher levels of IL-5 and lower levels of TGF-β mRNA. These data suggest that local upregulation of IL-5 may lead to nasal polyp formation at specific sites in the face of diffuse mucosal inflammation.

TGF-β, present in high amounts in regenerating epithelial cells, plays an essential role in the tissue remodeling process by regulating structural or inflammatory cell activation, proliferation and differentiation, and the deposition of extracellular matrix proteins [14, 15]. Many previous papers in western countries have reported that patients with CRSsNP showed higher levels of TGF-β expression, whereas those with CRSwNP showed lower TGF-β expression levels compared with the control subjects [6, 14, 16, 17]. However, there was no significant difference in TGF-β between our ECRS and non-ECRS groups. Thus, TGF-β may not be a suitable biomarker for classification of CRS patients.

Paranasal sinus mucosa from the ECRS patients demonstrated increased IL-5 expression in all harvested sites as compared with those from non-ECRS patients. In addition, the frontal recess mucosa of ECRS patients showed a significant increase in IL-5 compared with ethmoid sinus mucosa of ECRS patients. This result is quite innovative because significant ethmoid sinus eosinophilic inflammation is characteristic of ECRS patients and predominant opacification of the ethmoid sinus is one of the criteria of ECRS. IL-5 is pivotal for the recruitment and survival of eosinophils [14]. Thus, the frontal recess mucosa of the ECRS group is likely to be immensely influenced by persistent eosinophilic inflammation, to the same or greater extent as the ethmoid sinus. Therefore, the finding provides us with a rationale for the development of treatment strategies targeting eosinophilic inflammatory responses in the frontal sinus of ECRS patients.

Increased levels of IL-5 mRNA expression or protein production has been previously reported in the paranasal sinus mucosa of CRSwNP patients [6, 13, 18, 19]. Although our results in the frontal recess cannot be directly compared with the previous reports, both CRSwNP and ECRS are characterized by severe eosinophilic inflammation and may be based on similar pathogenic mechanisms.

IL-6 is a proinflammatory Th2 type cytokine that stimulates fibroblast proliferation and collagen synthesis. IL-6 is produced by a variety of cells including T and B lymphocytes, macrophages, eosinophils, epithelial cells, and fibroblasts [20]. In this study, a significant increase in IL-6 expression was noted in the frontal recess mucosa in the ECRS group as compared with the ethmoid sinus mucosa and nasal polyp in the ECRS group. In contrast, IL-6 expression in the frontal recess mucosa was not significantly different compared with the ethmoid sinus mucosa and nasal polyp in non-ECRS group. Thus, IL-6 expression may be associated with the pathophysiology of frontal sinusitis in ECRS patients and mechanisms of frontal eosinophilic sinusitis that differ from non-ECRS patients.

Exhaled NO measurements have become a standardized, reliable, and objective tool in the diagnosis and management of airway eosinophilic inflammation [21]. Interestingly, the human paranasal sinuses are a sizable source of intrinsic NO production, although the origin of nasal NO measured from human nasal airways has been a matter of debate [22]. We have previously reported that higher nasal NO levels in ECRS patients were closely correlated with augmented iNOS expression in response to proinflammatory cytokines and were also accompanied by the excretion of NO metabolites into the sinus mucosa [3].

In this study, the frontal recess and ethmoid sinus mucosa in the ECRS patients did not show a significant difference in iNOS expression as compared with the non-ECRS patients. This result may be due to the patient selection and sample size, or may reflect unimpaired ciliary clearance function in the non-ECRS patients with a sizable NO production. Further evidence, including local NO measurements for each paranasal sinus, is needed for a better understanding of the regulatory mechanisms of NO in ECRS.

## Conclusions

This study revealed that the cytokine expression profiles in the frontal recess mucosa in ECRS and non-ECRS patients are distinct. We therefore need to keep in mind the underlying eosinophilic inflammation that may cause intractable aggravation of mucosal diseases in the post-operative follow-up period when we treat frontal sinusitis in ECRS patients.

## Abbreviations

CRS: Chronic rhinosinusitis
CRSsNP: Chronic rhinosinusitis without nasal polyp
CRSwNP: Chronic rhinosinusitis with nasal polyp
CT: Computed tomography
ECRS: Eosinophilic chronic rhinosinusitis
ESS: Endoscopic sinus surgery
HE: Hematoxylin-eosin
IgE: Immunoglobulin E
IL-5: Interleukin-5
iNOS: Inducible nitric oxide synthase
JESREC: Japanese Epidemiological Survey of Refractory Eosinophilic Chronic Rhinosinusitis study
MLP: Modified Lothrop procedure
mRNA: messenger RNA
RAST: Radioallergosorbent test
SAE: *Staphylococcus* enterotoxin
TGF-β: Transforming growth factor-β

## References

[1] Han JK, Ghanem T, Lee B, Gross CW (2009). Various causes for frontal sinus obstruction. Am J Otolaryngol.

[2] Bassiouni A, Wolmald PJ (2013). Role of frontal sinus surgery in nasal polyp recurrence. Laryngoscope.

[3] Snidvongs K, Chin D, Sachs R, Earls P, Harvey RJ (2013). Eosinophilic rhinosinusitis is not a disease of ostiomeatal occlusion. Laryngoscope.

[4] Tokunaga T, Sakashita M, Haruna T, Asaka D, Takeno S, Ikeda H (2015). Novel scoring system and algorithm for classifying chronic rhinosinusitis: the JESREC Study. Allergy.

[5] Shi LL, Xiong P, Zhang L, Cao PP, Liao B, Lu X (2013). Features of airway remodeling in different types of Chinese chronic rhinosinusitis are associated with inflammation patterns. Allergy.

[6] Sejima T, Holtappels G, Kikuchi H, Imayoshi S, Ichimura K, Bachert C (2012). Cytokine profiles in Japanese patients with chronic rhinosinusitis. Allergol Int.

[7] Taruya T, Takeno S, Kubota K, Sasaki A, Ishino T, Hirakawa K (2015). Comparison of arginase isoform expression in patients with different subtypes of chronic rhinosinusitis. J Laryngol Otol.

[8] Takeno S, Taruya T, Ueda T, Noda N, Hirakawa K (2013). Increased exhaled nitric oxide and its oxidation metabolism in eosinophilic chronic rhinosinusitis. Auris Nasus Larynx.

[9] Lund VJ, Mackay IS (1993). Staging in rhinosinusitis. Rhinology.

[10] Tran KN, Beule AG, Singal D, Wormald PJ (2007). Frontal ostium restenosis after the endoscopic modified Lothrop procedure. Laryngoscope.

[11] Kubota K, Takeno S, Hirakawa K (2015). Frontal recess anatomy in Japanese subjects and its effect on the development of frontal sinusitis: computed tomography analysis. J Otolaryngol Head Neck Surg.

[12] Van Zele T, Claeys S, Gevaert P, Van Maele G, Holtappels G, Van Cauwenberge P (2006). Differentiation of chronic sinus diseases by measurement of inflammatory mediators. Allergy.

[13] Cao PP, Li HB, Wang BF, Wang SB, You XJ, Cui YH (2009). Distinct immunopathologic characteristics of various types of chronic rhinosinusitis in adult Chinese. J Allergy Clin Immunol.

[14] Otto BA, Wenzel SE (2008). The role of cytokines in chronic rhinosinusitis with nasal polyps. Curr Opin Otolaryngol Head Neck Surg.

[15] Watelet JB, Claeys C, Perez-Novo C, Gevaert P, Van Cauwenberge P, Bachert C (2004). Transforming growth factor β1 in nasal remodeling: differences between chronic rhinosinusitis and nasal polyposis. Am J Rhinol.

[16] Pezato R, Balsalobre L, Lima M, Bezerra TFP, Voegels RL, Gregorio LC (2013). Convergence of two major pathophysiologic mechanisms in nasal polyposis: immune response to staphylococcus aureus. J Otolaryngol Head Neck Surg.

[17] Kou W, Hu GH, Yao HB, Wang XQ, Shen Y, Kang HY (2012). Regulation of transforming growth factor-β1 activation and expression in the tissue remodeling involved in chronic rhinosinusitis. ORL J Otorhinolaryngol Relat Spec.

[18] Ba L, Du J, Liu F, Yang F, Han M, Liu S (2015). Distinct inflammatory profiles in atopic and nonatopic patients with chronic rhinosinusitis accompanied by nasal polyps in western China. Allergy Asthma Immunol Res.

[19] Hamilos DL, Leung DY, Wood R, Cunningham L, Bean DK, Yasruel Z (1995). Evidence for distinct cytokine expression in allergic versus nonallergic chronic sinusitis. J Allergy Clin Immunol.

[20] Ghaffer O, Lavigne F, Kamil A, Renzi P, Hamid Q (1998). Interleukin-6 expression in chronic sinusitis: colonization of gene transcripts to eosinophils, macrophages, T lymphocytes, and mast cells. Otolaryngol Head Neck Surg.

[21] Taylor DR, Pijnenburg MW, Smith AD, DeJongste JC (2006). Exhaled nitric oxide measurements: clinical application and interpretation. Thorax.

[22] Ragab SM, Lund VJ, Saleh HA, Scadding G (2006). Nasal nitric oxide in objective evaluation of chronic rhinosinusitis therapy. Allergy.

